# Long‐Term Benefit of Thalamic Deep Brain Stimulation in POLR3A Mutation‐Associated Action Tremor

**DOI:** 10.1002/mdc3.70002

**Published:** 2025-02-20

**Authors:** Martina Minnerop, Alisha Reinhardt, Petyo Nikolov, Bahne H. Bahners, Julian Caspers, Julia Gelenar Marae, Christian J. Hartmann, Stefan J. Groiss, Katrin Amunts, Jan Vesper, Alfons Schnitzler

**Affiliations:** ^1^ Institute of Neuroscience and Medicine (INM‐1) Research Center Jülich Jülich Germany; ^2^ Institute of Clinical Neuroscience and Medical Psychology Medical Faculty & University Hospital Düsseldorf, Heinrich Heine University Düsseldorf Düsseldorf Germany; ^3^ Department of Neurology, Center for Movement Disorders and Neuromodulation Medical Faculty & University Hospital Düsseldorf, Heinrich‐Heine‐University Düsseldorf Düsseldorf Germany; ^4^ Center for Brain Circuit Therapeutics, Department of Neurology Brigham & Women's Hospital, Harvard Medical School Boston Massachusetts USA; ^5^ Department of Diagnostic and Interventional Radiology Medical Faculty & University Hospital Düsseldorf, Heinrich‐Heine‐Universität Düsseldorf Düsseldorf Germany; ^6^ Neurocenter Düsseldorf Düsseldorf Germany; ^7^ C. and O. Vogt Institute for Brain Research, Medical Faculty & University Hospital Düsseldorf, Germany Heinrich Heine University Düsseldorf Düsseldorf Germany; ^8^ Department of Functional Neurosurgery and Stereotaxy, Neurosurgical Clinic, Medical Faculty Heinrich Heine University Düsseldorf Düsseldorf Germany

**Keywords:** VIM, DBS, POLR3A, tremor, leukodystrophy

The intronic heterozygous c.1909 + 22G>A POLR3A mutation in combination with a null allele cause adolescent‐onset spastic ataxia with action tremor, sensory disturbances, and dental problems [1]. About 59% of published cases present with tremor, mostly of upper limb (72%, see Data S1). Here, we report sustained tremor improvement after deep brain stimulation (DBS) over a five‐year‐follow‐up period in one of these patients included in [1] (Case F3‐1).

The 71‐year‐old male, offspring of German non‐consanguineous parents, reported progressive gait disturbances since age 15 (wheelchair‐bound since age 46), dysarthria, impaired fine motor coordination, restless legs syndrome, and occasionally urinary urge incontinence. At age 60, he developed a progressively disabling action tremor, preventing writing or using a computer and requiring feeding during meals.

He presented with pronounced spastic paraplegia, reduced deep tendon reflexes, extensor plantar response, and ankle contractures. Cerebellar signs included saccadic smooth pursuit, hypometric saccades, impaired suppression of the oculocephalic reflex and cerebellar dysarthria. The complex motor phenotype encompassed bilateral upper and lower limb ataxia/dysmetria with an irregular, coarse(3‐4 Hz) action tremor with intention tremor‐component of the proximal upper limbs, trunk, and head (“yes”‐tremor).

In the *Scale for the Assessment and Rating of Ataxia* (SARA [2]) he reached 33/40 points. Both legs had distal hypesthesia and diminished vibration sense. Somatosensory and motor‐evoked potentials were abnormal. Nerve conduction studies revealed sensory‐motor neuropathy (see Supplementary Material). MRI revealed mild generalized, but no specific cerebellar atrophy (Fig. 1D), and bilateral T2‐hyperintensities along the superior cerebellar peduncles (SCPs) with a T1‐hypointense correlate (Fig. 1A–C), indicating secondary myelin degradation, as described for other patients with hypomorphic POLR3A mutations [1].

**Figure 1:**
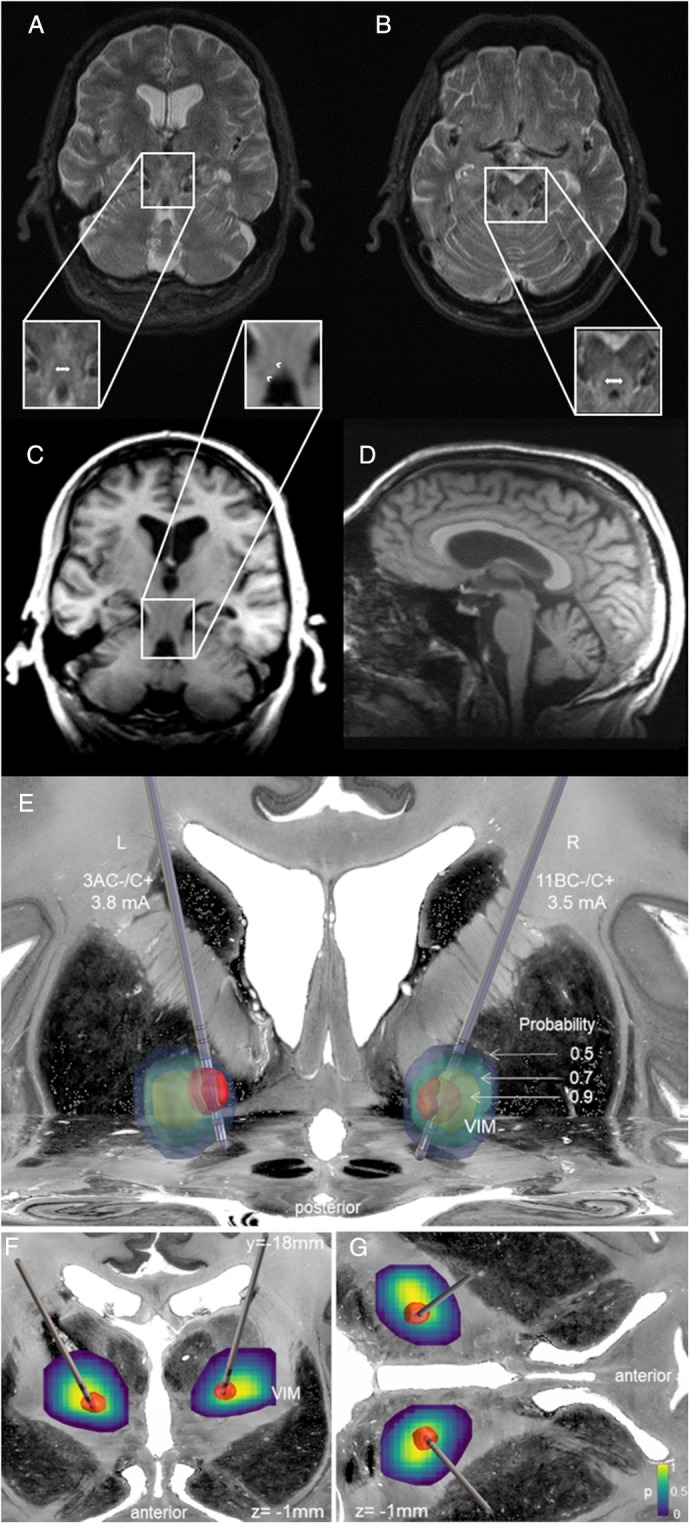
T2‐weighted MR‐images in a paracoronal (A) and axial view (B). T1‐weighted images in a paracoronal (C) and sagittal view (D). White double‐arrow (A, B) shows bilateral SCP hyperintensity with a hypointense correlate in T1‐weighted images (arrow heads in C). (E–G) 3D‐visualization of the DBS electrodes and the potential distribution (red sphere around the electrodes) created with *Lead‐DBS v3.0* (https://www.lead‐dbs.org/). For visualization, the *Big Brain Model* was used as backdrop image and the probabilistic histological *Julich Brain Atlas* for VIM 3D‐rendering (https://julich‐brain‐atlas.de/) (E) illustrates the DBS electrodes and potential distribution in relation to the extent and localization of the VIM at probability thresholds of 0.5, 0.7, and 0.9. In (B, C) the electrodes are shown in relation to section planes through the volume of activated tissue with the respective plane of the probabilistic VIM atlas as heatmap on top illustrating continuous probabilities ranging from 0 to 1.

Since medication showed no benefit or was contraindicated (see Data S1), the patient underwent DBS of the thalamic ventral intermediate nucleus (VIM) using directional leads (Abbott SJM Infinity, Abbott, Texas, USA). Standard directional monopolar high‐frequency stimulation (2A−/C+, 2.8 mA, 60 μs, 150 Hz) was chosen for the left, and omnidirectional modality (10ABC−/C+, 2.4 mA, 60 μs, 130 Hz) for the right VIM.

The DBS led to clear improvement (Video S1) relevant to the patient's everyday life: The tremor of the upper limbs, head and trunk were markedly reduced (see Data S1). The patient could again eat independently with a spoon, spread bread with a knife, and use the computer. Adaptation of the stimulation parameters were required over time (left: 3 AC−/C+, 3.8 mA, 90 μs, 160 Hz; right: 11 BC−/C+, 3.5 mA, 60 μs, 150 Hz; Fig. 1E–G), but the DBS‐benefit still persisted at a five‐year‐follow‐up, also reflected in the SARA [2] (DBS‐OFF/ON 38 vs. 34.5/40 points), and the *Essential Tremor Rating Assessment Scale* ([3], DBS‐OFF/ON: 96 vs. 80.5/99.5 points).

This benefit is remarkable since the T2‐hyperintense SCPs belong to the dentato‐rubro thalamic‐tract, important for tremor control by VIM‐DBS [4]. However, the cause of tremor in POLR3A patients appears complex, as not all patients with SCP‐T2‐hyperintensity present with tremor [1]. Thus, the functional impact of the T2‐hyperintense (demyelinated) SCPs for the dentato‐rubro‐thalamic‐tract and its role in the occurrence of tremor in these patients is unclear and requires further investigations.

DBS in patients suffering from POLR3A‐related disorders has currently only been described for one adult patient with cognitive decline and parkinsonism, successfully treated with pallidal DBS [5]. Due to the profound and long‐lasting benefit relevant to everyday life our case illustrates, that patients with action tremor due to POLR3A mutations should be considered suitable candidates for DBS.

## Author Roles

(1) Research project: A. Conception, B. Organization, C. Execution; (2) Statistical Analysis: A. Design, B. Execution, C. Review and Critique; (3) Manuscript Preparation: A. Writing of the first draft, B. Review and Critique.

M.M.: 1A, 1B, 1C, 3A, 3B.

A.R.: 1C, 3A, 3B.

P.N.: 1B, 1C, 3B.

B.H.B.: 1C, 3B.

J.C.: 1B, 1C, 3B.

J.G.M.: 1B, 1C, 3B.

C.J.H.: 1B, 1C, 3B.

S.J.G.: 1B, 1C, 3B.

K.A.: 1A, 1B, 3B.

J.V.: 1B, 1C, 3B.

A.S.: 1A, 1B, 3B.

## Disclosure

**Ethical Compliance Statement:** The study was approved by the Ethics Committee of the Medical Faculty of the University of Düsseldorf, Germany (No. 2022–1807).

**Funding Sources and Conflict of Interest:** This project received funding from the Helmholtz Association's Initiative and Networking Fund through the Helmholtz International BigBrain Analytics and Learning Laboratory (HIBALL) under the Helmholtz International Lab grant agreement InterLabs‐0015 and from the Joint Lab “Supercomputing and Modeling of the Human Brain”. The authors declare that there are no conflicts of interest relevant to this work.

**Financial Disclosures for the Previous 12 Months:** MM was supported by the Deutsche Forschungsgemeinschaft (MI 709/2–1) and by the German Heredo Ataxia Society (DHAG) and received honoraria from Biogen, unrelated to this research. AR gratefully acknowledges the Friedrich‐Ebert‐Foundation for its support (doctoral scholarship). PN received travel expenses from Abbott Medical and Boston Scientific, manufacturers of DBS devices. SJG received consulting fees/ honoraria unrelated to this research from Abbott, Abbvie, Bial and Inomed. BHB gratefully acknowledges support by the Prof. Dr. Klaus Thiemann Foundation (Parkinson Fellowship 2022). KA had received funding from the European Union's Horizon 2020 Research and Innovation Programme under Grant No. 101147319 (EBRAINS 2.0 Project). JV received consulting fees unrelated to this research from Abbott, Boston Scientific, Medtronic. Unrelated to this research, he received speaker honoraria from bsh medical communication, Abbott, Boston Scientific, Novartis. AS was supported by the Deutsche Forschungsgemeinschaft (TRR 295), unrelated to this research. He received consulting fees unrelated to this research from Abbott, Zambon, and Abbvie. Unrelated to this research, he received speaker honoraria from bsh medical communication, Abbott, Kyowa Kirin, Novartis, Abbvie, and Alexion. The authors JC, JGM, CJH declare that there are no additional disclosures to report.

**Declaration of patient consent:** Written informed consent was obtained from the patient. Additionally, the patient gave separate consent for audio, video and photo recordings.

## Supporting information


**Video S1.** Clinical benefit of DBS with regard to the tremor in the patient at the age of 74 years, at two‐years‐follow‐up (left OFF, right ON DBS).

**Data S1.** Additional information on the clinical phenotype in compound‐heterozygous carriers of the intronic POLR3A variant; Additional case‐related clinical information; Detailed case‐related results of the electrophysiological examination; Case‐related clinical (side‐) effects of DBS.

## Data Availability

The data that support the findings of this study are available on request from the corresponding author. The data are not publicly available due to privacy or ethical restrictions.
