# Supplementary material for: Long‐Term Benefit of Thalamic Deep Brain Stimulation in POLR3A Mutation‐Associated Action Tremor
Source: Mov Disord Clin Pract. 2025 Feb 20;12(6):882–4. doi: 10.1002/mdc3.70002 (PMC12187964; doi:10.1002/mdc3.70002)
Supplement: Supplementary file 2 — Data S1. Additional information on the clinical phenotype in compound‐heterozygous carriers of the intronic POLR3A variant; Additional case‐related clinical information; Detailed case‐related results of the electrophysiological examination; Case‐related clinical (side‐) effects of DBS. [file MDC3-12-882-s001.docx]

**Long-term Benefit of Thalamic Deep Brain Stimulation in POLR3A mutation-associated Action Tremor**

**Supplemental clinical information**

Additional information on the clinical phenotype in compound-heterozygous carriers of the intronic POLR3A variant

Biallelic POLR3A mutations frequently cause autosomal recessive-inherited spastic ataxia, accounting for about 3% of hitherto genetically unclassified autosomal recessive and sporadic cases [1]. About 80% of these patients are heterozygous carrier of the intronic variant Heterozygous c.1909+22G>A, POLR3A mutations associated with in combination with a null allele cause adolescent-onset spastic ataxia with action tremor, sensory disturbances, and dental problems such as hypodontia and early loss of teeth due to severe and aggressive periodontal disease at a young age [1]. To date (Literature search on PubMed 30 April 2024), 73 cases with this intronic variant have been reported [1,6-16], often associated with limb or head/voice tremor (43/73, 59%), with upper limb tremor being the most common tremor manifestation (31/43, 72%).

1. Minnerop M, Kurzwelly D, Wagner H, et al.Hypomorphic mutations in POLR3A are a frequent cause of sporadic and recessive spastic ataxia. Brain 2017; 140: 1561-1578.

6. Rydning SL, Koht J, Sheng Y, et al. Biallelic POL3A variants confirmed as a frequent cause of hereditary ataxia and spastic paraparesis. Brain 2019; 142: e12.

7. de Assis Pereira Matos PCA, Gama MTD, Bezerra MLE, da Rocha AJ, BArsottini OGP, Pedroso JL. *POL3A-*Related Disorder Presenting with Late-Onset Dystonia and Spastic Paraplegia. Mov Disord Clin Pract 2020; 7: 467-469.

8. Infante J, Serrano-Cárdenas KM, Corral-Juan M, et al. POLR3A-related spastic ataxia: new mutations and a look into the phenotype. J Neurol. 2020;267:324-330.

9. Morales-Rosado JA, Macke EL, Cousin MA, Oliver GR, Dhamija R, Klee EW. Interpretation challenges of novel dual-class missense and splice-impacting variant in POL3A-related late-onset hereditary spastic ataxia. Mol Genet Genomic Med 2020; 8: e1341.

10. Ruggiero L, Iovino A, Dubbioso R, et al. Multimodal evaluation of an Italian family with a hereditary spastic paraplegia and POLR3A mutations. Ann Clin Transl Neurol. 2020;7:2326-2331.

11. Fellner A, Lossos A, Kogan E, et al. Two intronic cis-acting variants in both alleles of the POLR3A gene cause progressive spastic ataxia with hypodontia. Clin Genet. 2021;99:713-718.

12. Baviera-Muñoz R, Carretero-Vilarroig L, Vázquez-Costa JF et al. Diagnostic Efficacy of Genetic Studies in a Series of Hereditary Cerebellar Ataxias in Eastern Spain. Neurol Genet. 2022;8:e200038.

13. Di Donato I, Gallo A, Ricca I, et al. POL3A variants in hereditary spastic paraparesis and ataxia: clinical, genetic, and neuroradiological findings in a cohort of Italian patients. Neurol Sci 2022; 43: 1071-1077.

14. Systma TM, Chen DH, Rolf B, et al. Spinal-cord predominant neuropathology in adult-onset case of POL3A-related spastic ataxia. Neuropathology 2022; 42: 58-65.

15. Zea Vera A, Bruce A, Larsh TR, et al. Spectrum of Pediatric to Early Adulthood POLR3A-Associated Movement Disorders. Mov Disord Clin Pract. 2022;10:316-322.

16. McKenna MC, O'Connor A, Lockhart A, et al. POL3A-related disorders: expanding the clinical phenotype. J Neurol 2024; 271: 3635-3638.

Additional case-related clinical information

The patient had cardiovascular risk factors, including a non-insulin-dependent diabetes mellitus, recurrent basaliomas/squamous cell carcinoma, and a non-Hodgkin lymphoma in the past. His sister and half-siblings were reported healthy.

Myelon imaging was not performed. The severe head tremor made any tomographic imaging of the (cervical) spinal cord or brain difficult and required sedation.

Since medication with either primidone (500mg/day) or clonazepam (6-18mg/day) showed no benefit and beta-blockers were contraindicated due to the patient’s history of asthma and diabetes mellitus, the patient underwent DBS of the thalamic ventral intermediate nucleus (VIM) using directional leads (Abbott SJM Infinity, Abbott, Texas, USA).

Detailed case-related results of the electrophysiological examination

By transcranial magnetic stimulation elicited motor evoked potentials of the first dorsal interosseous muscle revealed prolonged total and central motor conduction latencies (total – R/L: 32.7/33.4ms (normal range ≤24.5ms); central - R/L: 15.5/15.7ms (normal range ≤8.7ms)). Motor-evoked potentials of the tibialis anterior muscle (only investigated after cortical stimulation) as well as cortical somatosensory evoked potentials after stimulating the medial and tibial nerve were bilaterally absent. Nerve conduction studies revealed sensory-motor, axonal-demyelinating neuropathy with prolonged distal motor latencies and prolonged F-wave latencies of the right median nerve, reduced amplitude of the compound muscle action potential and reduced motor conduction time of the left peroneal nerve (after stimulation above the fibula head), absent F-wave of the right peroneal nerve and absent sensory nerve action potential of the right ulnar nerve. Motor conduction study of the left tibial nerve, and sensory nerve conduction studies of the right median and the left sural nerve were normal.

Case-related clinical (side-) effects of DBS

The patient experienced as stimulation-side effect a worsening of his dysarthria during DBS-ON, which further deteriorated with increasing stimulation intensities and was not diminished by reducing pulse width, while on the other hand DBS-OFF did only lead to small improvement. In accordance with the patient’s wishes, pulse width was therefore optimized for tremor suppression. Furthermore, a postoperatively increased spasticity required antispastic medication with baclofen, which in turn was discontinued because of an increase of proximal paraparesis with loss of some residual ability of hip flexion, not affecting his already wheelchair-bound motor abilities. However, this loss of hip flexion did not improve again after discontinuation of the medication. It remains unclear, whether this should be regarded as side effect of an internal capsule stimulation; other factors like lesion-related side effects, postoperative immobilization or progression of disease might have contributed to the postoperative increase of the paraparesis as well.

The benefit regarding tremor and tremor-related hypermetria was reflected in the SARA scores [2] over the follow-up period, which showed a sustained benefit of four points during DBS-ON compared to DBS-OFF (after two years: OFF/ON 31vs.27, after five years: 38vs.34.5/40 points), despite slight differences due to assessment by different investigators. Additionally, the *Essential Tremor Rating Assessment Scale* (TETRAS, [3]) was used at the five-years follow-up, further demonstrating the benefit of DBS (OFF/ON: 96 vs. 80.5/99.5 points), with clear relevance also for activities of daily living (items: drinking from a glas, writing, using the computer mouse as the most affected task).
